# Supplementary material for: Comparative Proteomic Analysis of Exosomes Derived from Patients Infected with Non-Tuberculous Mycobacterium and Mycobacterium tuberculosis
Source: Microorganisms. 2023 Sep 17;11(9):2334. doi: 10.3390/microorganisms11092334 (PMC10535683; doi:10.3390/microorganisms11092334)
Supplement: Supplementary file 1 [file microorganisms-11-02334-s001.zip › microorganisms-2594369-supplementary.pdf]

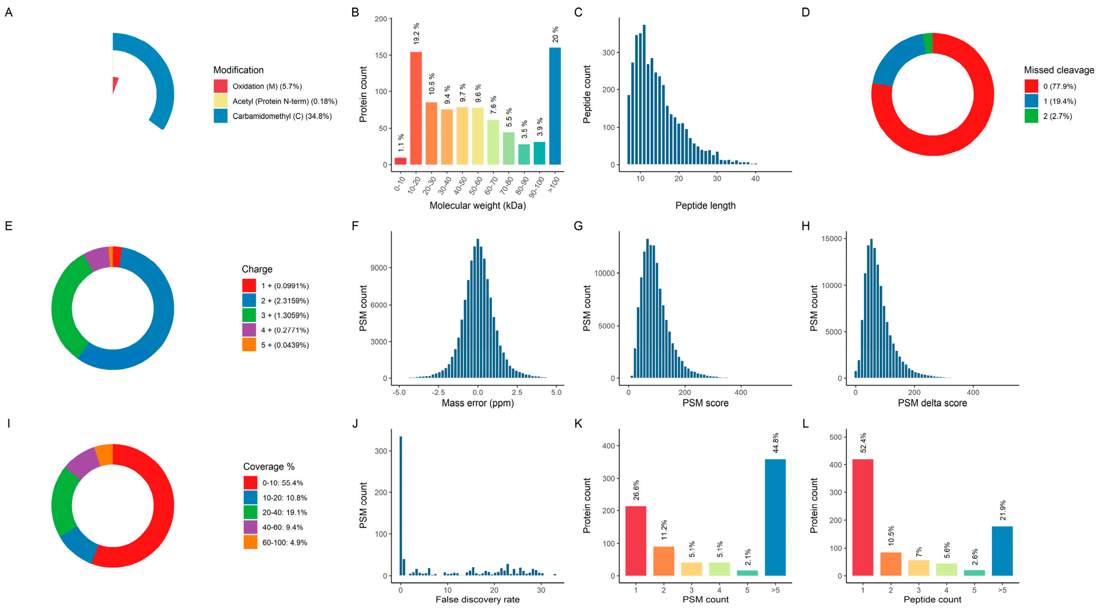

Figure S1 Quality control information. (A) Distribution of peptide modifications. (B) Distribution of protein molecular weight (kDa). (C) Peptide segment length distribution diagram. (D) Distribution of the number of missed peptide cuts. (E) Peptide charge distribution diagram. (F) Distribution of mass error. (G) Distribution of peptide-spectrum matches (PSM) score. (H) Distribution of PSM delta score. (I) Distribution of protein identification coverage. (J) FDR distribution of peptides. (K) Distribution of the number of proteins identified to the spectrum. (L) Distribution of the number of proteins identified to peptides.

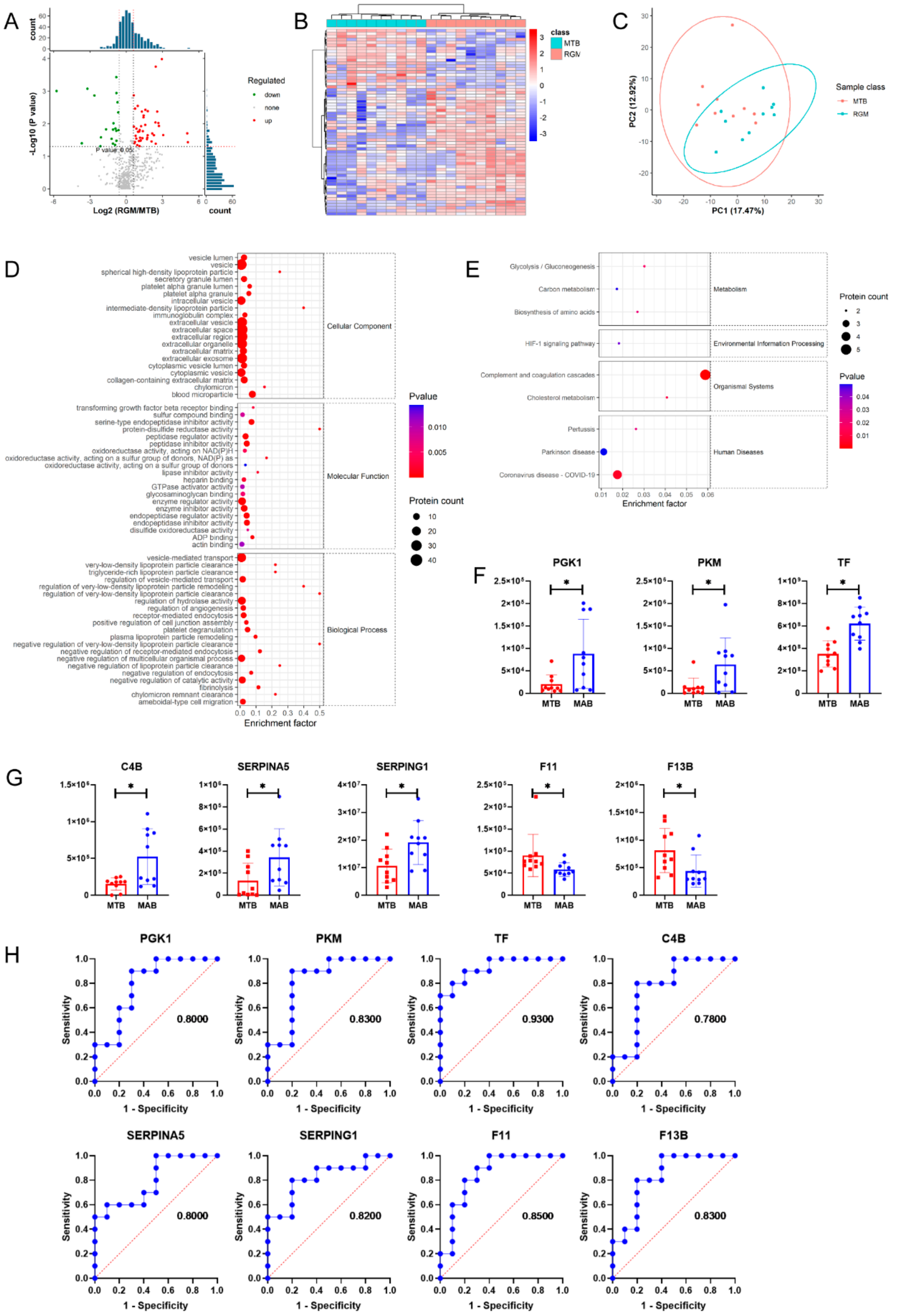

Figure S2 Proteomics analysis of plasma exosomes between MAB group and MTB group. (A) Volcano plot of differential expression proteins. (B) Heatmap of differential expression proteins. (C) PCA plot of expression plasma exosomal proteins. (D) GO term enrichment map of differential expression proteins. (E) KEGG pathway enrichment map of differential expression proteins. (F) Histogram of differential protein expression in gluconeogenesis. (G) Histogram of differential protein expression in complement and coagulation cascades. (H) ROC analysis of complement and coagulation cascades differential expression proteins.

* $p < 0.05$.

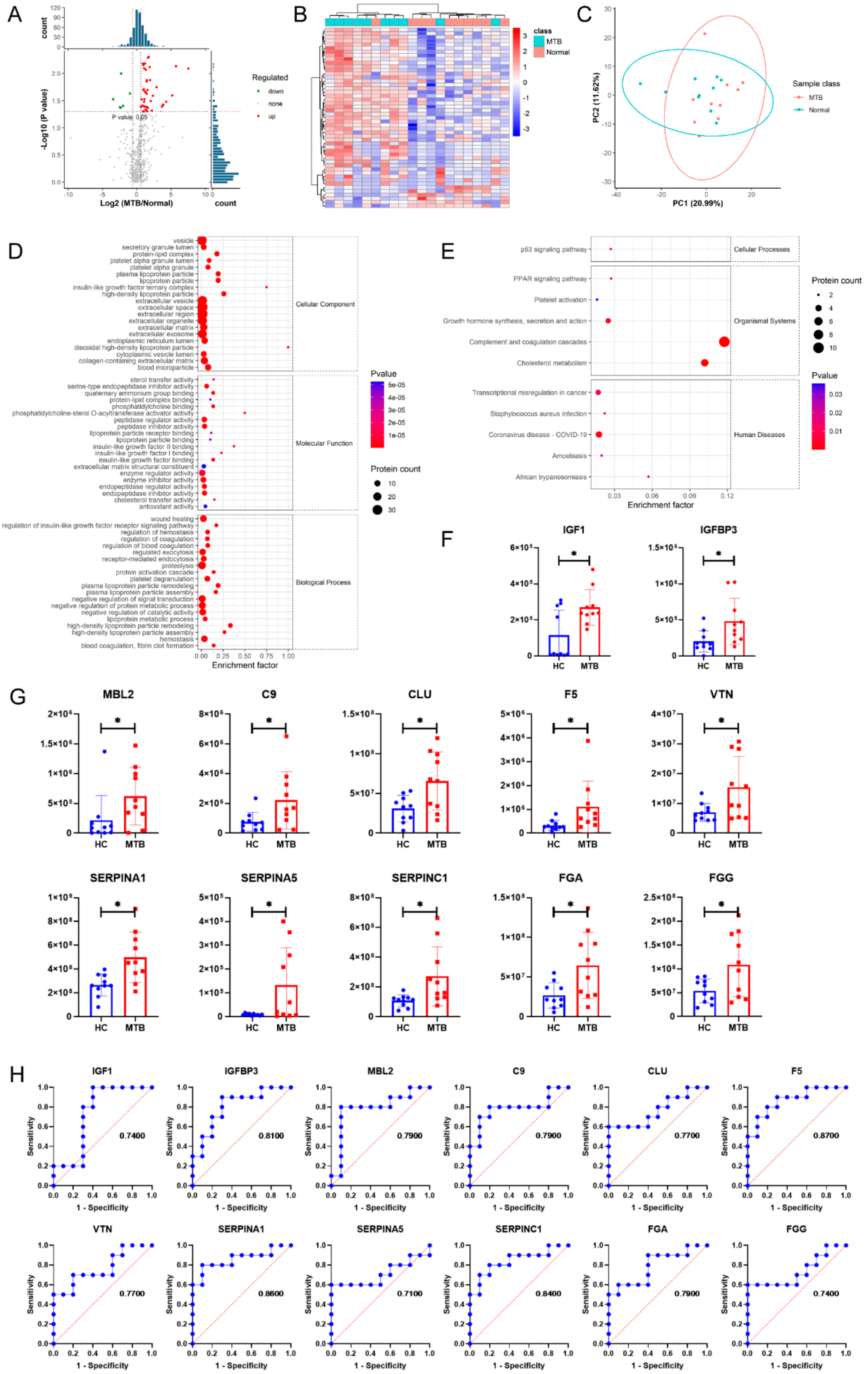

Figure S3 Proteomics analysis of plasma exosomes between active MTB-infected patients and healthy controls. (A) Volcano plot of differential expression plasma exosomal proteins. (B) Heatmap of differential expression plasma exosomal proteins. (C) PCA

plot of expression plasma exosomal proteins. (D) GO term enrichment map of differential expression plasma exosomal proteins. (E) KEGG pathway enrichment map of differential expression plasma exosomal proteins. (F) Histogram of differential protein expression in p53 signaling pathway. (G) Histogram of differential protein expression in complement and coagulation cascade. (H) ROC analysis of differential expression exosomal proteins. * $p < 0.05$.

Table S1 Characteristics of patients and healthy controls.

**Participant characteristics**	**HC**	**MAB**	**MAC**	**MTB**
Number (n)	10	10	10	10
Age, years	37.10±9.37	56.20±10.65	59.80±10.16	31.1±3.78
Male (n)	5	1	4	2
Female (n)	5	9	6	8

Age is presented as Mean ± SD. n, number of subjects.
